# Age-related collagen turnover of the interstitial matrix and basement membrane: Implications of age- and sex-dependent remodeling of the extracellular matrix

**DOI:** 10.1371/journal.pone.0194458

**Published:** 2018-03-29

**Authors:** Stephanie N. Kehlet, Nicholas Willumsen, Gabriele Armbrecht, Roswitha Dietzel, Susanne Brix, Kim Henriksen, Morten A. Karsdal

**Affiliations:** 1 Nordic Bioscience A/S, Herlev, Denmark; 2 DTU Bioengineering, Department of Biotechnology and Biomedicine, Technical University of Denmark, Kgs. Lyngby, Denmark; 3 Center for Muscle and Bone Research, Department of Radiology, Campus Benjamin Franklin, CHARITE – University Medicine Berlin, Berlin, Germany; Oregon Health and Science University, UNITED STATES

## Abstract

The extracellular matrix (ECM) plays a vital role in maintaining normal tissue function. Collagens are major components of the ECM and there is a tight equilibrium between degradation and formation of these proteins ensuring tissue health and homeostasis. As a consequence of tissue turnover, small collagen fragments are released into the circulation, which act as important biomarkers in the study of certain tissue-related remodeling factors in health and disease. The aim of this study was to establish an age-related collagen turnover profile of the main collagens of the interstitial matrix (type I and III collagen) and basement membrane (type IV collagen) in healthy men and women.

By using well-characterized competitive ELISA-assays, we assessed specific fragments of degraded (C1M, C3M, C4M) and formed (PINP, Pro-C3, P4NP7S) type I, III and IV collagen in serum from 617 healthy men and women ranging in ages from 22 to 86. Subjects were divided into 5-year age groups according to their sex and age. Groups were compared using Kruskal-Wallis adjusted for Dunn’s multiple comparisons test and Mann-Whitney t-test. Age-specific changes in collagen turnover was most profound for type I collagen. PINP levels decreased in men with advancing age, whereas in women, the level decreased in early adulthood followed by an increase around the age of menopause (age 40–60). Sex-specific changes in type I, III and IV collagen turnover was present at the age around menopause (age 40–60) with women having an increased turnover. In summary, collagen turnover is affected by age and sex with the interstitial matrix and the basement membrane being differently regulated. The observed changes needs to be accounted for when measuring ECM related biomarkers in clinical studies.

## Introduction

The extracellular matrix (ECM) is the backbone of all tissues. It is composed of several structural proteins, including collagens, which play a vital role for the function and maintenance of normal tissue function. Collagen type I and III are the most abundant collagens of the interstitial matrix and essential for its structure. The basement membrane, underlying epithelial or endothelial cells, primarily consist of collagen type IV which ensure optimal cell polarization and function [1,2].

ECM tissue turnover, i.e. the tight balance between protein degradation and formation can be classified as two processes: 1) tissue modeling, occurring during development and growth where new tissue is being generated; 2) tissue remodeling, where functional tissue is being maintained by replacing old and damaged proteins with new ones [3,4]. Every healthy organ is undergoing continuous remodeling with a tight control between degradation and formation. However, this delicate balance might be disturbed, leading to connective tissue disorders such as fibrosis and cancer [5]. Measurements of ECM turnover products in blood have shown that circulating components of the ECM, especially collagens, are elevated in fibrotic diseases [6–10] and cancer [11,12]. We have developed a panel of serum-based assays specifically measuring collagen fragments that reflect either degradation or formation separately [13–18]. The principle behind these assays is the use of monoclonal antibodies exclusively reacting with a specific fragment of a certain protein which become exposed after specific protease-mediated degradation. Antibodies raised against pro-peptides of pro-collagens reflect collagen formation whereas antibodies recognizing small neo-epitopes on peptides derived from collagen degradation of the triple helical region represent collagen degradation [5,19,20]. Table 1 summarizes the assays we have used in this study and which ECM process they represent.

**Table 1:** Description of the collagen degradation and formation assays used in this study.

Biomarker	Specification	Process	Surrogate measure
C1M[14]	Neo-epitope of MMP-2,9,13 mediated degradation of type I collagen	Type I collagen degradation	Chronic inflammation
PINP[13]	Internal epitope in the N-terminal pro-peptide of type I collagen	Type I collagen formation	Primarily bone synthesis
C3M[16]	Neo-epitope of MMP-9 mediated degradation of type III collagen	Type III collagen degradation	Chronic inflammation
Pro-C3[15]	Released N-terminal pro-peptide of type III collagen	Type III collagen formation	Fibrosis
C4M[18]	Neo-epitope of MMP-2,9,12 mediated degradation of type IV collagen alpha 1	Type IV collagen degradation	Chronic inflammation
P4NP7S[17]	Internal epitope in the 7S domain of type IV collagen	Type IV collagen formation	Fibrosis

PINP, which measures the N-terminal pro-peptide released during collagen formation, have been showed to primarily reflect synthesis of bone matrix [21]. C1M, which measures a MMP-degraded fragment of type I collagen released during tissue remodeling, is closely related to chronic inflammation with high levels being present in various inflammatory diseases [14,22–25]. Pro-C3 measures the pro-peptide of type III collagen, i.e. synthesis, and C3M measures a MMP-generated type III collagen fragment, i.e. degradation. Increased levels of Pro-C3 and C3M have also been linked to inflammatory diseases and especially fibrosis [25–30]. P4NP7S, which reflects type IV collagen formation by measuring the 7S domain of type IV collagen, has been associated with fibrosis of the liver [31] and C4M, which reflects MMP-mediated degradation of the basement membrane, is elevated in patients with diseases displaying chronic inflammation [18,24,32–34].

While it has been shown that levels of ECM proteins vary significantly with age [35–39], a collagen turnover profile in men and women of varying age using a well-defined panel of markers directly measuring degradation and formation of interstitial matrix and basement membrane collagens has never been publihsed. In this study, we investigated age- and sex-dependent ECM turnover as function of age in healthy men and women by measuring biomarkers of formation and degradation of the most abundant collagens of the interstitial matrix (collagen type I and III) and basement membrane (collagen type IV) in serum.

## Materials and methods

### Serum samples

The serum samples used in this study originated from a population-based cross-sectional study where subjects were recruited in 2007–2011 from a random sample of all districts in Berlin provided by the resident registration office. A total number of 617 subjects, comprising 303 healthy men and 314 healthy women aged 22–86, were included in this study. Characteristics of the study population are presented in Table 2.

**Table 2:** Basal characteristics of the study population.

**Men (n = 303)**				
**Age group**	**Age (median, years)** **Range (min.-max.)**	**Weight (median, kg)** **Range (min.-max.)**	**Height (median, cm)** **Range (min.-max.)**	**BMI (median, kg/m**^**2**^**)** **Range (min.-max.)**
**20–24 (n = 19)**	24 22–24	75 58–128	181 170–197	23.1 19.8-37-4
**25–29 (n = 25)**	26 25–29	76 61–104	179 168–202	23.8 20.5–30.0
**30–34 (n = 24)**	31 30–34	78 64–120	178 164–189	24.7 18.7–35.8
**35–39 (n = 24)**	36 35–37	83 64–120	180 167–193	26.1 19.0–38.0
**40–44 (n = 21)**	43 40–44	87 53–122	179 164–195	26.5 19.7–36.4
**45–49 (n = 18)**	48 45–49	85 69–101	178 169–199	26.2 22.0–30.4
**50–54 (n = 24)**	52 50–54	83 57–106	181 171–188	25.5 17.1–32.4
**55–59 (n = 28)**	57 55–59	54 57–101	177 159–192	27.0 20.7–34.5
**60–64 (n = 23)**	63 60–64	82 70–109	176 166–197	26.1 22.9–34.9
**65–69 (n = 30)**	67 65–69	83 50–126	172 161–183	27.1 17.8–38.7
**70–74 (n = 20)**	72 70–74	84 67–102	175 158–184	29.0 22.2–33.6
**75–79 (n = 17)**	79 77–79	77 60–118	169 159–183	27.0 23.9–36.4
**80+ (n = 30)**	82 80–86	78 52–99	175 161–182	25.8 16.1–30.8
**Women (n = 314)**				
**Age group**	**Age (median, years)** **Range (min.-max.)**	**Weight (median, kg)** **Range (min.-max.)**	**Height (median, cm)** **Range (min.-max.)**	**BMI (median, kg/m**^**2**^**)** **Range (min.-max.)**
**20–24 (n = 12)**	23 22–24	63 48–105	173 158–184	21.7 18.6–33.1
**25–29 (n = 29)**	26 25–29	62 52–74	166 157–180	21.4 18.5–27.9
**30–34 (n = 22)**	32 30–34	65 46–90	168 154–186	22.2 19.4–31.9
**35–39 (n = 25)**	36 35–37	72 51–93	164 155–185	25.1 18.8–34.0
**40–44 (n = 26)**	43 40–44	69 50–89	168 157–183	23.7 17.7–33.6
**45–49 (n = 24)**	48 45–49	76 51–95	166 146–180	26.8 18.5–36.1
**50–54 (n = 25)**	52 50–54	66 52–104	165 156–173	23.1 20.8–36.4
**55–59 (n = 26)**	57 55–59	66 50–87	163 155–175	25.4 19.1–32.0
**60–64 (n = 32)**	62 60–64	67 53–109	161 154–172	26.1 20.5–38.9
**65–69 (n = 29)**	67 65–69	64 47–97	161 152–171	24.9 19.2–33.6
**70–74 (n = 20)**	72 70–74	69 53–79	159 146–175	27.0 21.6–31.7
**75–79 (n = 21)**	78 75–79	70 56–108	160 147–171	26.5 21.7–38.3
**80+ (n = 23)**	81 80–86	64 46–79	159 147–177	25.3 20.1–31.7

Venous blood samples were collected by trained medical technologists between 08:00 and 10:00 AM after a 12 h fasting period. 30 min. after blood drawing, samples were centrifuged at 3500 rpm for 10 min, serum was obtained and samples were stored at -80°C until analysis.

The study was approved by the local ethics committee (Ethikkommission der Charité, Charité –Universitätsmedizin Berlin, EA4/095/05) as well as the German Radiation Protection Ordinance (Z5-22462/2-2005-063). Written informed consent was obtained from all participants before they were included into the study and the study was carried out in accordance with ICH-GCP and according to the Declaration of Helsinki.

### Collagen turnover biomarker analysis

All biomarker assays were manufactured by Nordic Bioscience (Herlev Denmark) and performed according to the manufacturer’s specifications. The assays are competitive ELISA’s which have been thoroughly validated for their use in human serum samples and technically characterized with regards to linearity, accuracy and reproducibility (the original assay reference for each biomarker is listed in Table 1). To eliminate/reduce inter-assay variation, all biochemical markers were measured using a single lot of reagents and serum controls were included on each plate. Females and males, as well as age-groups were randomly distributed on the different ELISA plates. All samples were analyzed by a CAP/CLIA-accredited laboratory (Nordic Bioscience Laboratory, Herlev, Denmark).

The neo-epitope biomarkers of matrix metalloproteinase (MMP) degraded type I, type III and type IV collagen (C1M, C3M, C4M) and type I, type III and type IV collagen formation products (PINP, Pro-C3, P4NP7S) were assessed in serum as previously described [13–18]. Briefly, 96-well pre-coated streptavidin plates were coated with biotinylated synthetic peptides specific for the protein of interest and incubated for 30 minutes at 20°C. 20 μL of standard peptide or pre-diluted serum sample were added to designated wells followed by the addition of peroxidase-conjugated specific monoclonal antibodies and incubated for 1 h at 20°C or overnight at 4°C. Finally, tetramethylbenzinidine (TMB) (cat. 438OH, Kem-En-Tec Diagnostics, Denmark) was added to each well and the plates were incubated for 15 minutes at 20°C. All incubation steps included shaking at 300 rpm and after each incubation step, the plates were washed five times with wash buffer (20 mM Tris, 50 mM NaCl, pH 7.2). The enzymatic reaction was stopped by adding 0.18 M H_2_SO_4_ and absorbance was measured at 450 nm with 650 nm as reference. A calibration curve was plotted using a 4-parameter logistic curve fit.

### Statistical analysis

Subjects were stratified into 5-year interval age groups. To investigate if the biomarkers changed with age, the levels of the individual biomarkers in each 5-year age group were compared using a Kruskal—Wallis ANOVA (S1 Table). The p-values were adjusted to account for multiple comparisons using Dunnett´s method. An increase or decrease is defined as a significant difference between two following age groups, e.g. age group 25–29 vs. 30–34 (two adjacent age groups).

To investigate if the biomarkers varied according to sex, the level of the individual biomarkers between men and women in the five-year age groups was compared using the Mann-Whitney test. Serum biomarker data can be found in S1 Data. Graph design and statistical analyses were performed using GraphPad Prism version 7.03 (GraphPad Software, Inc., CA, USA).

## Results

### Age specific changes in collagen remodeling

#### Type I collagen

**Fig 1:**
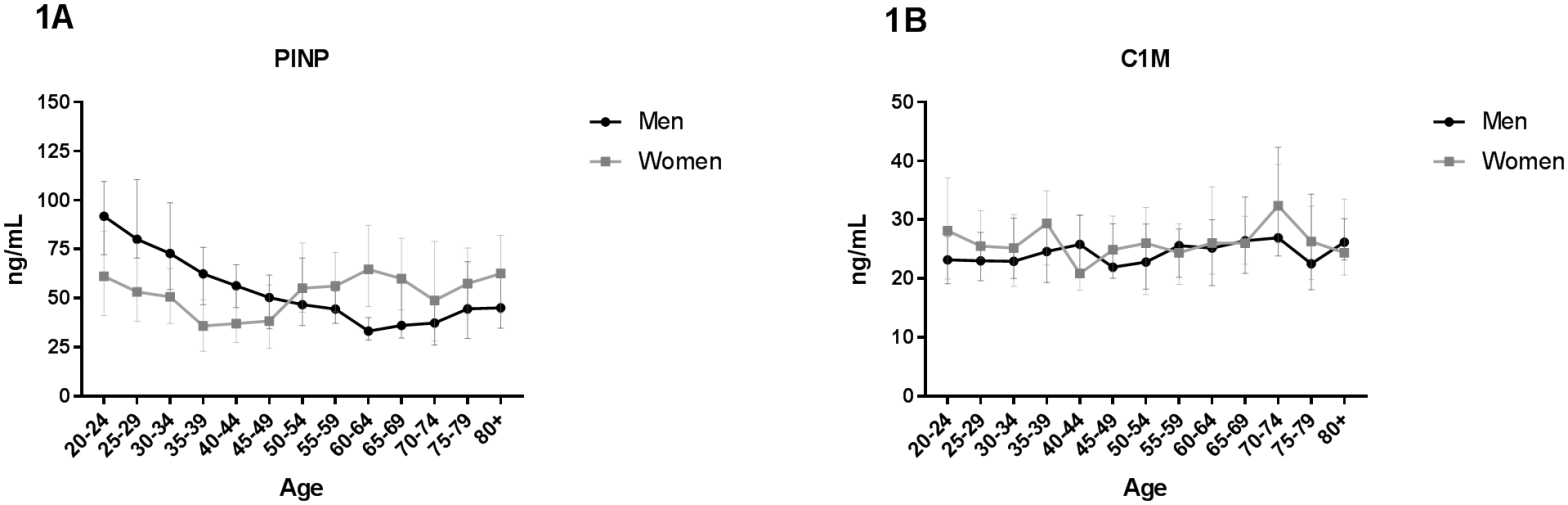
Type I collagen turnover as function of age. Biomarkers reflecting degradation and formation of type I collagen were measured in serum from 303 healthy men and 314 healthy women aged 22–86 divided into 5-year age groups. (A) Formation of interstitial type I collagen (PINP) and (B) degradation of interstitial type I collagen (C1M). Statistical significance of C1M and PINP between each age group was calculated using ANOVA comparing the mean of each group with the mean of every other group and is presented at the different age groups in S1 Table. All data are shown as median and interquartile range.

Type I collagen formation and degradation were measured with the PINP and C1M assays, respectively. In men, the highest level of PINP was found at age 20–24 (Fig 1A).

The level declined with increasing age. Comparing the level at age 20–24 with the rest of the age-groups, a significant decrease was observed from age 45–49 to 80+ (S1 Table). In women, the highest level of PINP was found at age 20–24 and 80+. PINP levels decreased from age 20–24 until age 35–39, but not significantly (Fig 1A). Around menopause (average age 40–60 [40]) the level significantly increased until age 60–64 (S1 Table).

The level of C1M in men was relatively stable with no significant changes (Fig 1B). The level of C1M in women was also relatively stable with only a significant increase between age group 40–44 and 70–74 (Fig 1B) (S1 Table).

#### Type III collagen

**Fig 2:**
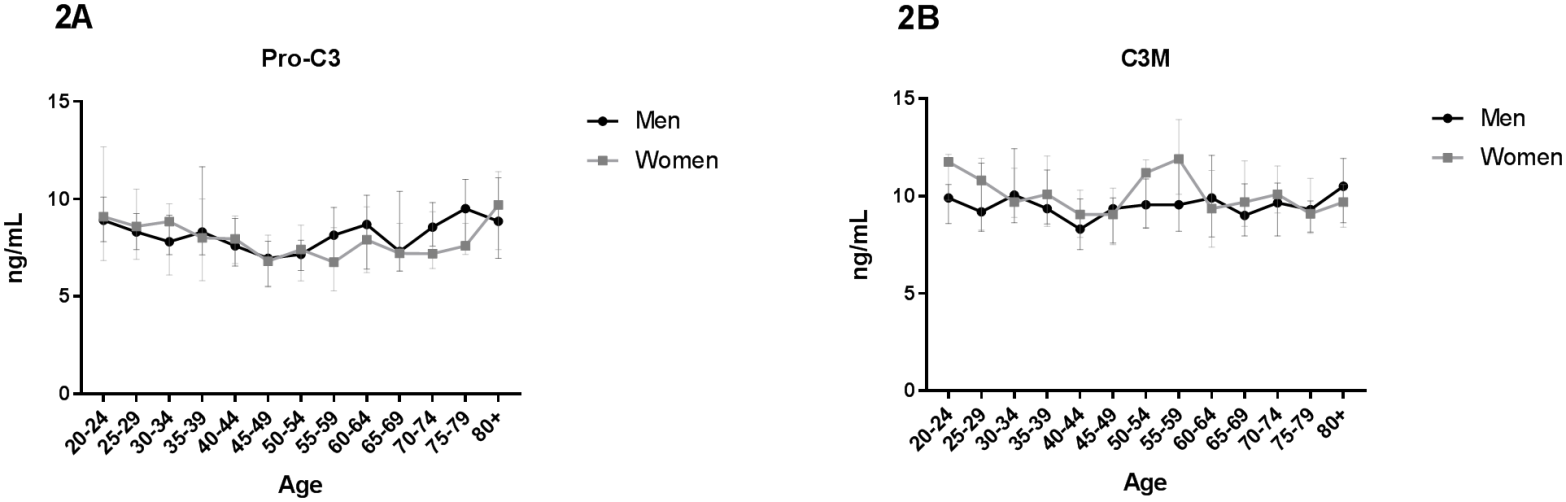
Type III collagen turnover as function of age. Biomarkers reflecting degradation and formation of type III collagen were measured in serum from 303 healthy men and 314 healthy women aged 22–86 divided into 5-year age groups. (A) Formation of interstitial type III collagen (Pro-C3) and (B) degradation of interstitial type III collagen (C3M). Statistical significance of C3M and Pro-C3 between each age group was calculated using ANOVA comparing the mean of each group with the mean of every other group and is presented at the different age groups in S1 Table. All data are shown as median and interquartile range.

The ELISA assays Pro-C3 and C3M were used to measure type III collagen formation and degradation, respectively. Pro-C3 levels in men showed a significant decrease between age 20–24 and 45–49 followed by a significant increase until age 80+ (Fig 2A) (S1 Table).

The level of Pro-C3 was relatively stable in women with no significant changes until age 45–49. A significant increase from age 45–49 and 55–59 until age 80+ was observed (Fig 2A) (S1 Table).

The level of C3M in men was relatively stable from age 20–80+ with no significant changes (Fig 2B). The same pattern was seen in women (Fig 2B). However, around menopause (average age 40–60 [40]) the level significantly increased until age 55–59 (S1 Table) followed by a steep significant decline (S1 Table).

#### Type IV collagen

**Fig 3:**
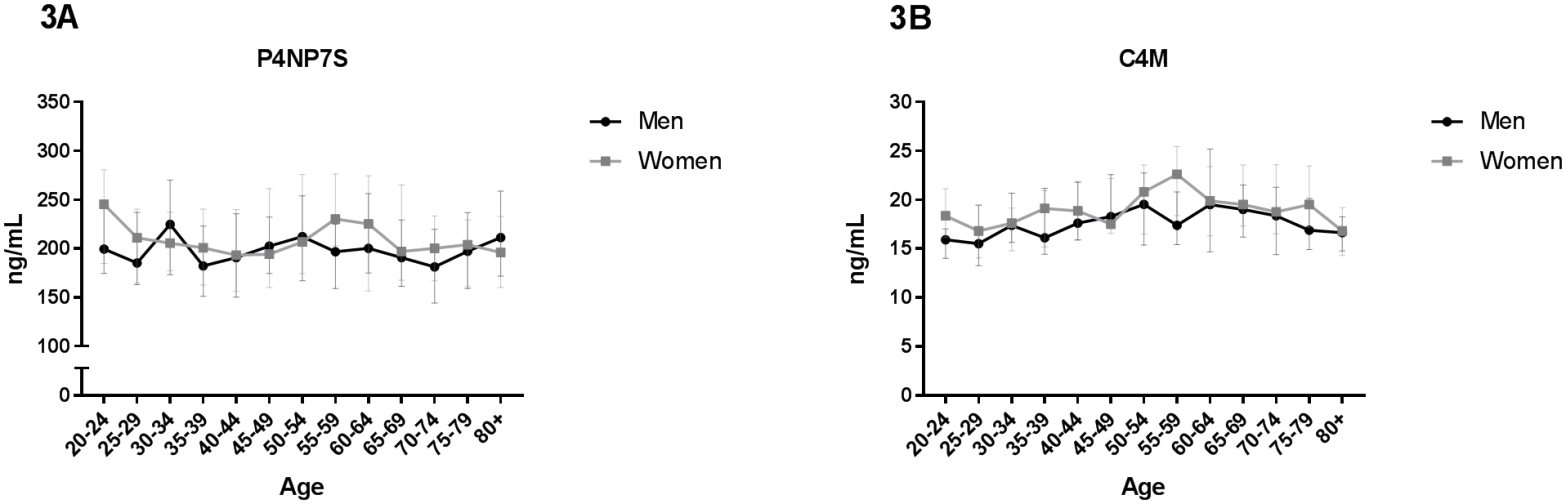
Type IV collagen turnover as function of age. Biomarkers reflecting degradation and formation of type IV collagen were measured in serum from 303 healthy men and 314 healthy women aged 22–86 divided into 5-year age groups. (A) Formation of basement membrane type IV collagen (P4NP7S) and (B) degradation of basement membrane type IV collagen (C4M). Statistical significance of C4M and P4NP7S between each age group was calculated using ANOVA comparing the mean of each group with the mean of every other group and is presented at the different age groups in S1 Table. All data are shown as median and interquartile range.

Type IV collagen formation and degradation were measured with the P4NP7S and C4M assays, respectively. The level of P4NP7S in men and women was relatively stable with no significant changes across the age span (Fig 3A).

The same pattern was observed with C4M in men (Fig 3B). In women, a significant increase was observed from age 25–29 reaching a maximum at age 55–59 (Fig 1F) (S1 Table). The level significantly decreased reaching its starting level at age 80+ (S1 Table).

### Sex specific changes in collagen remodeling

#### Type I collagen

For PINP, the starting level was significantly higher in men compared to women until the age of 45–49 (Table 3).

**Table 3:** Sex-specific changes in collagen remodeling.

	**PINP**				**C1M**			
**Age group**	**Median (men)**	**Median (women)**	**Significant**	**P-value**	**Median (men)**	**Median (women)**	**Significant**	**P-value**
**20–24**	91.8	61.2	Yes	0.02	23.2	28.2	No	0.18
**25–29**	80.1	53.2	Yes	0.0004	23.0	25.5	No	0.05
**30–34**	72.9	50.7	Yes	0.001	23.0	25.2	No	0.84
**35–39**	62.5	35.8	Yes	0.0004	24.6	29.4	No	0.11
**40–44**	56.3	37.0	Yes	0.001	25.8	20.9	No	0.25
**45–49**	50.3	38.4	No	0.18	22.0	24.9	No	0.38
**50–54**	46.7	55.0	No	0.34	22.8	26.0	No	0.82
**55–59**	44.4	56.2	Yes	0.01	25.6	24.4	No	0.85
**60–64**	33.2	64.7	Yes	<0.0001	25.2	26.1	No	0.66
**65–69**	36.1	59.9	Yes	0.001	26.5	26.0	No	0.62
**70–74**	37.3	48.9	No	0.19	27.0	32.4	No	0.27
**75–79**	44.6	57.4	No	0.10	22.5	26.3	No	0.67
**80+**	45.1	62.5	Yes	0.02	26.2	24.4	No	0.90
	**PRO-C3**				**C3M**			
**Age group**	**Median (men)**	**Median (women)**	**Significant**	**P-value**	**Median (men)**	**Median (women)**	**Significant**	**P-value**
**20–24**	8.9	9.1	No	0.80	9.9	11.8	No	0.07
**25–29**	8.3	8.6	No	0.66	9.2	10.8	No	0.21
**30–34**	7.8	8.9	No	0.85	10.1	9.7	No	0.68
**35–39**	8.3	8.0	No	0.25	9.4	10.1	No	0.30
**40–44**	7.6	8.0	No	0.87	8.3	9.1	No	0.33
**45–49**	7.0	6.8	No	0.94	9.4	9.1	No	0.78
**50–54**	7.2	7.4	No	0.70	9.6	11.2	No	0.24
**55–59**	8.2	6.8	Yes	0.03	9.6	11.9	Yes	0.02
**60–64**	8.7	7.9	No	0.38	9.9	9.4	No	0.49
**65–69**	7.3	7.2	No	0.31	9.0	9.7	No	0.20
**70–74**	8.9	7.2	Yes	0.04	9.7	10.1	No	0.25
**75–79**	9.5	7.6	Yes	0.01	9.3	9.1	No	0.64
**80+**	8.9	9.7	No	0.29	10.5	9.7	No	0.34
	**P4NP7S**				**C4M**			
**Age group**	**Median (men)**	**Median (women)**	**Significant**	**P-value**	**Median (men)**	**Median (women)**	**Significant**	**P-value**
**20–24**	199.5	245.2	No	0.20	15.9	18.4	Yes	0.04
**25–29**	185.3	210.9	No	0.49	15.5	16.8	No	0.56
**30–34**	224.9	205.6	No	0.52	17.4	17.6	No	0.89
**35–39**	182.3	200.8	No	0.27	16.1	19.1	No	0.50
**40–44**	190.6	193.3	No	0.82	17.6	18.9	No	0.70
**45–49**	202.5	194.2	No	0.72	18.3	17.5	No	0.36
**50–54**	212.3	206.8	No	0.77	19.6	20.8	No	0.58
**55–59**	196.7	230.3	Yes	0.02	17.4	22.6	Yes	0.001
**60–64**	200.3	225.2	No	0.88	19.5	19.9	No	1.00
**65–69**	190.7	196.9	No	0.31	19.0	19.5	No	0.25
**70–74**	199.5	245.2	No	0.20	15.9	18.4	Yes	0.04
**75–79**	185.3	210.9	No	0.49	15.5	16.8	No	0.56
**80+**	224.9	205.6	No	0.52	17.4	17.6	No	0.89

The level of the individual biomarkers between men and women in the five-year age groups was compared using the Mann-Whitney test. P-values are indicated and represent significant difference (p≤0.05) between men and women.

Around this age, the level starts to increase in women, whereas the level in men continues to decrease. In the postmenopausal period (age 60–80+), the level was significantly higher in women compared to men, except at the age of 70–79 (Table 3).

For C1M, no significant difference between the sexes was observed.

#### Type III collagen

The level of Pro-C3 was significantly different in women compared to men in specific age groups in the end of the menopausal period (age 55–59) and the postmenopausal period (70–74 and 75–79) with men having the highest level (Table 3).

For C3M, a significantly higher level was observed in women as compared to men at the age of 55–59 (menopausal period) (Table 3).

#### Type IV collagen

As with C3M, a significantly higher level of P4NP7S in women at age 55–59 was observed (Table 3). The level of C4M was significant different in men and women at age 20–24 and 55–59 with women having the highest level (Table 3).

## Discussion

The present study defines a collagen turnover profile of the main collagens of the interstitial matrix (type I and III collagen) and basement membrane (type IV collagen) as function of age in healthy men and women. The main findings of the study were: 1) age specific changes in collagen turnover was most profound for type I collagen with its formation being strongly age-dependent and 2) sex specific changes in collagen turnover was most apparent during the menopausal and post-menopausal periods with the interstitial matrix and basement membrane being differently regulated. To our knowledge, this is the first time a collagen turnover profile measuring collagen formation and degradation separately, has been established in healthy men and women across the age span of adults.

Type I collagen is the main ECM component of bone, composing 90% of the organic matrix [41] and its formation seems to be strongly age-dependent (Fig 1A). The level of PINP was higher in young men and women compared to older subjects with a progressive decrease until the age around 50. This observation is in accordance with previous studies showing a decrease in women between age 30–35, stable level from age 35–45 followed by an increase until the age of 65 [35,37]. In men, a significant decrease with age has also been observed [37]. The decline in PINP levels until middle-age may reflect a shift from bone modeling (formation) in childhood and early adulthood to bone remodeling (maintenance). As seen in Fig 1B, degradation of type I collagen (C1M) is relatively stable throughout the study period. As C1M is a marker of tissue inflammation and does not represent bone degradation [14], this may explain why C1M is not age-dependent and PINP is. One may argue that elder subjects have more inflammation than younger, hence a rise in C1M late in life was expected. However, the observed results may reflect that the elder subjects in this study are “super” healthy compared to the “normal” elder population.

Type III collagen is one of the main interstitial collagens and structurally similar to type I collagen. Despite being often associated with type I collagen, the formation of type III collagen showed a different pattern with an increase in both men and women after age 45–49 and 55–59 respectively. As Pro-C3 is a marker of fibrosis [15,29,42], these results suggest that tissue fibrosis increases with advancing age.

Type IV collagen is one of the most abundant components of the basement membrane. No association between age and biomarker level (P4NP7S and C4M) was observed, however C4M in women was significantly increased with time comparing age 25–29 with age 55–59 (Fig 3B, S1 Table). These data suggest that basement membrane degradation is increased in the menopausal period.

Karsdal *et al*.[3] have conducted a study similar to this in rats. They found that type I collagen had an increased turnover in younger rats compared to old rats consistent with our findings. Type III collagen turnover was not significantly influenced by age while type IV collagen degradation was slightly upregulated in younger animals. These results can be translated into the present data in humans which is of importance when conducting pre-clinical and clinical studies in ECM remodeling diseases.

To investigate sex-specific changes in collagen turnover, the level of the individual biomarkers between men and women in the five-year age groups was compared. C3M, P4NP7S and C4M showed a significant difference between men and women in the menopausal period (age 40–60) with women having a higher level of collagen remodeling. PINP and Pro-C3 displayed an increase in women compared to men in the post-menopausal period (after the age of 60). PINP showed a significant lower level in women throughout the pre-menopausal period (before the age of 40). Together, these data suggest that hormonal status might affect collagen remodeling. Whether this is related to estrogen levels or other menopausal mechanisms needs further investigations. However, the fact that the biomarkers decreases again during the post-menopausal period indicates that a specific event during menopause is somehow influencing the collagen turnover. These sex-specific changes in collagen turnover could be associated with diseases that is more prevalent in postmenopausal women, as with osteoporosis and PINP [43].

One limitation of this study is the lack of younger subjects. Studies measuring the aminoterminal pro-peptide of procollagen type III have shown a high level in young children which decreased with advancing age with a short increase around puberty [38,39]. These data may reflect the process of modeling where new tissue is being formed during growth whereas the results of the current study only reflects the end of this process involving a shift towards ECM remodeling. Another limitation is the lack of information on the subject’s renal function. We are currently investigating the association between renal function and the collagen turnover biomarkers. Unpublished data from our group show no association between renal function and C1M, PINP, C3M, PRO-C3 and C4M.

Subjects with ECM remodeling disorders, such as fibrosis and cancer, have been shown to have a different ECM turnover compared to healthy individuals [18,22,25,29,33,44] and attention has been drawn towards the involvement of matrix proteins in ECM remodeling disorders [45,46]. For example in cancer, it is now becoming evident that the tumor milieu, i.e. the ECM, is just as important as the tumor itself for tumor progression and metastasis [47,48]. The ability to measure changes in ECM turnover in ECM-involved pathologies, could be an important step towards a better personalized medicine [49,50].

The presented data are important to consider when conducting clinical studies focusing on ECM-related disorders as these biomarkers have been shown to associate with various connective tissue disorders where the ECM balance is skewed [5,15,18,22,25,29,33,44].

## Conclusions

In conclusion, collagen turnover is affected by age and sex with the interstitial matrix (type I and III collagen) and the basement membrane (type IV collagen) being differently regulated. We have established an age- and sex-dependent collagen turnover profile in healthy men and women and the observed changes needs to be accounted for when measuring ECM related biomarkers.

## Supporting information

S1 Table Age-related changes in collagen remodeling. Statistical significance (p-value) for each biomarker calculated using ANOVA comparing the mean of each group with the mean of every other group. Ns refers to no significance. The following biomarkers showed no significance and are therefore not presented: C1M (men), C3M (men), P4NP7S (men and women) and C4M (men).
(DOCX)

S1 Data Serum biomarker data for each subject. (XLSX)

## References

[1] Yurchenco PD, Schittny JC. Molecular architecture of basement membranes. FASEB J [Internet]. 1990;4(6):1577–90. Available from: http://www.fasebj.org/content/4/6/1577.short%5Cnhttp://www.ncbi.nlm.nih.gov/pubmed/2180767 10.1096/fasebj.4.6.2180767 2180767

[2] Bosman FT, Stamenkovic I. Functional structure and composition of the extracellular matrix. J Pathol [Internet]. 2003;200(4):423–8. Available from: http://www.ncbi.nlm.nih.gov/pubmed/12845610%5Cnhttp://doi.wiley.com/10.1002/path.1437 10.1002/path.1437 12845610

[3] Karsdal MA, Genovese F, Madsen EA, Manon-Jensen T, Schuppan D. Collagen and tissue turnover as a function of age: Implications for fibrosis. J Hepatol. 2016;64(1):103–9. doi: 10.1016/j.jhep.2015.08.014 26307398 10.1016/j.jhep.2015.08.014

[4] Frantz C, Stewart KM, Weaver VM. The extracellular matrix at a glance. J Cell Sci [Internet]. 2010;123(24):4195–200. Available from: http://jcs.biologists.org/cgi/doi/10.1242/jcs.023820 10.1242/jcs.023820 PMC2995612 21123617

[5] Karsdal MA, Nielsen MJ, Sand JM, Henriksen K, Genovese F, Bay-Jensen AC, et al Extracellular matrix remodeling: the common denominator in connective tissue diseases. Possibilities for evaluation and current understanding of the matrix as more than a passive architecture, but a key player in tissue failure. Vol. 11, Assay.Drug Dev.Technol. p. 70–92. 10.1089/adt.2012.474 PMC3593693 23046407

[6] Patel K, Shackel NA. Current status of fibrosis markers. Curr Opin Gastroenterol [Internet]. 2014;30(3):253–9. Available from: http://content.wkhealth.com/linkback/openurl?sid=WKPTLP:landingpage&an=00001574-201405000-00007 10.1097/MOG.0000000000000059 24671009

[7] Rosenberg WMC, Voelker M, Thiel R, Becka M, Burt A, Schuppan D, et al Serum markers detect the presence of liver fibrosis: A cohort study. Gastroenterology. 2004;127(6):1704–13. 15578508 10.1053/j.gastro.2004.08.052

[8] Forel JM, Guervilly C, Hraiech S, Voillet F, Thomas G, Somma C, et al Type III procollagen is a reliable marker of ARDS-associated lung fibroproliferation. Intensive Care Med. 2015;41(1):1–11. doi: 10.1007/s00134-014-3524-0 25354475 10.1007/s00134-014-3524-0

[9] Biver E, Chopin F, Coiffier G, Brentano TF, Bouvard B, Garnero P, et al Bone turnover markers for osteoporotic status assessment? A systematic review of their diagnosis value at baseline in osteoporosis. Vol. 79, Joint Bone Spine. 2012 p. 20–5. 10.1016/j.jbspin.2011.05.003 21724445

[10] Lotz M, Martel-Pelletier J, Christiansen C, Brandi M-L, Bruyère O, Chapurlat R, et al Value of biomarkers in osteoarthritis: current status and perspectives. Ann Rheum Dis [Internet]. 2013 11 [cited 2017 Jun 22];72(11):1756–63. Available from: http://www.ncbi.nlm.nih.gov/pubmed/23897772 10.1136/annrheumdis-2013-203726 PMC3812859 23897772

[11] Hanke B, Wein a, Martus P, Riedel C, Voelker M, Hahn EG, et al Serum markers of matrix turnover as predictors for the evolution of colorectal cancer metastasis under chemotherapy. Br J Cancer [Internet]. 2003;88(8):1248–50. Available from: http://www.pubmedcentral.nih.gov/articlerender.fcgi?artid=2747566&tool=pmcentrez&rendertype=abstract 10.1038/sj.bjc.6600832 PMC2747566 12698191

[12] Kang CY, Wang J, Axell-House D, Soni P, Chu M-L, Chipitsyna G, et al Clinical Significance of Serum COL6A3 in Pancreatic Ductal Adenocarcinoma. J Gastrointest Surg [Internet]. 2014 1 4 [cited 2017 Jun 22];18(1):7–15. Available from: http://www.ncbi.nlm.nih.gov/pubmed/24002763 10.1007/s11605-013-2326-y 24002763

[13] Leeming DJ, Larsen D V, Zhang C, Hi Y, Veidal SS, Nielsen RH, et al Enzyme-linked immunosorbent serum assays (ELISAs) for rat and human N-terminal pro-peptide of collagen type I (PINP)—assessment of corresponding epitopes. Clin Biochem [Internet]. 2010 10 [cited 2017 Jun 22];43(15):1249–56. Available from: http://linkinghub.elsevier.com/retrieve/pii/S0009912010003401 10.1016/j.clinbiochem.2010.07.025 20709044

[14] Leeming D, He Y, Veidal S, Nguyen Q, Larsen D, Koizumi M, et al A novel marker for assessment of liver matrix remodeling: An enzyme-linked immunosorbent assay (ELISA) detecting a MMP generated type I collagen neo-epitope (C1M). Biomarkers [Internet]. 2011;16(7):616–28. Available from: http://www.ncbi.nlm.nih.gov/pubmed/21988680 10.3109/1354750X.2011.620628 21988680

[15] Nielsen MJ, Nedergaard AF, Sun S, Veidal SS, Larsen L, Zheng Q, et al The neo-epitope specific PRO-C3 ELISA measures true formation of type III collagen associated with liver and muscle parameters. Am J Transl Res. 2013;5(3):303–15. 23634241 PMC3633973

[16] Barascuk N, Veidal SS, Larsen L, Larsen D V, Larsen MR, Wang J, et al A novel assay for extracellular matrix remodeling associated with liver fibrosis: An enzyme-linked immunosorbent assay (ELISA) for a MMP-9 proteolytically revealed neo-epitope of type III collagen. Clin Biochem. 2010;43(10–11):899–904. doi: 10.1016/j.clinbiochem.2010.03.012 20380828 10.1016/j.clinbiochem.2010.03.012

[17] Leeming DJ, Karsdal MA, Rasmussen LM, Scholze A, Tepel M. Association of Systemic Collagen Type IV Formation with Survival among Patients Undergoing Hemodialysis. Metze K, editor. PLoS One [Internet]. 2013 8 22 [cited 2017 Jun 22];8(8):e71050 Available from: http://www.ncbi.nlm.nih.gov/pubmed/23990924 10.1371/journal.pone.0071050 PMC3750054 23990924

[18] Sand JM, Larsen L, Hogaboam C, Martinez F, Han M, Røssel Larsen M, et al MMP Mediated Degradation of Type IV Collagen Alpha 1 and Alpha 3 Chains Reflects Basement Membrane Remodeling in Experimental and Clinical Fibrosis—Validation of Two Novel Biomarker Assays. Tsilibary EC, editor. PLoS One [Internet]. 2013 12 23 [cited 2017 Jun 22];8(12):e84934 Available from: http://www.ncbi.nlm.nih.gov/pubmed/24376856 10.1371/journal.pone.0084934 PMC3871599 24376856

[19] Karsdal MA, Henriksen K, Leeming DJ, Woodworth T, Vassiliadis E, Bay-Jensen AC. Novel combinations of Post-Translational Modification (PTM) neo-epitopes provide tissue-specific biochemical markers-are they the cause or the consequence of the disease? Vol. 43, Clinical Biochemistry. 2010 p. 793–804. 10.1016/j.clinbiochem.2010.03.015 20381482

[20] Karsdal MA, Bay-Jensen AC, Leeming DJ, Henriksen K, Christiansen C. Quantification of “end products” of tissue destruction in inflammation may reflect convergence of cytokine and signaling pathways–implications for modern clinical chemistry. Biomarkers [Internet]. 2013;18(5):375–8. Available from: http://www.tandfonline.com/doi/full/10.3109/1354750X.2013.789084 10.3109/1354750X.2013.789084 23721060

[21] Vasikaran S, Eastell R, Bruyère O, Foldes AJ, Garnero P, Griesmacher A, et al Markers of bone turnover for the prediction of fracture risk and monitoring of osteoporosis treatment: A need for international reference standards. Osteoporos Int. 2011;22(2):391–420. doi: 10.1007/s00198-010-1501-1 21184054 10.1007/s00198-010-1501-1

[22] Willumsen N, Bager CL, Leeming DJ, Smith V, Christiansen C, Karsdal MA, et al Serum biomarkers reflecting specific tumor tissue remodeling processes are valuable diagnostic tools for lung cancer. Cancer Med [Internet]. 2014;3(5):1136–45. Available from: http://www.pubmedcentral.nih.gov/articlerender.fcgi?artid=4302665&tool=pmcentrez&rendertype=abstract 10.1002/cam4.303 PMC4302665 25044252

[23] Siebuhr AS, Petersen KK, Arendt-Nielsen L, Egsgaard LL, Eskehave T, Christiansen C, et al Identification and characterisation of osteoarthritis patients with inflammation derived tissue turnover. Osteoarthr Cartil. 2014;22(1):44–50. doi: 10.1016/j.joca.2013.10.020 24216059 10.1016/j.joca.2013.10.020

[24] Leeming Leeming, Sand Jannie M., Nielsen Mette J., Genovese Martinez, et al Serological Investigation of the Collagen Degradation Profile of Patients with Chronic Obstructive Pulmonary Disease or Idiopathic Pulmonary Fibrosis. Biomark Insights [Internet]. 2012;119 Available from: http://la-press.com/serological-investigation-of-the-collagen-degradation-profile-of-patie-article-a3336 10.4137/BMI.S9415 PMC3448496 23012495

[25] Kehlet SN, Sanz-Pamplona R, Brix S, Leeming DJ, Karsdal MA, Moreno V. Excessive collagen turnover products are released during colorectal cancer progression and elevated in serum from metastatic colorectal cancer patients. Sci Rep [Internet]. 2016 11 28 [cited 2017 Aug 10];6(1):30599 Available from: http://www.ncbi.nlm.nih.gov/pubmed/27465284 10.1038/srep30599 PMC4964349 27465284

[26] Genovese F, Boor P, Papasotiriou M, Leeming DJ, Karsdal MA, Floege J. Turnover of type III collagen reflects disease severity and is associated with progression and microinflammation in patients with IgA nephropathy. Nephrol Dial Transplant. 2016;31(3):472–9. doi: 10.1093/ndt/gfv301 26311218 10.1093/ndt/gfv301

[27] Jenkins RG, Simpson JK, Saini G, Bentley JH, Russell AM, Braybrooke R, et al Longitudinal change in collagen degradation biomarkers in idiopathic pulmonary fibrosis: An analysis from the prospective, multicentre PROFILE study. Lancet Respir Med. 2015;3(6):462–72. doi: 10.1016/S2213-2600(15)00048-X 25770676 10.1016/S2213-2600(15)00048-X

[28] Leeming DJ, Karsdal MA, Byrjalsen I, Bendtsen F, Trebicka J, Nielsen MJ, et al Novel serological neo-epitope markers of extracellular matrix proteins for the detection of portal hypertension. Aliment Pharmacol Ther. 2013;38(9):1086–96. doi: 10.1111/apt.12484 24099470 10.1111/apt.12484 PMC3935409

[29] Nielsen MJ, Kazankov K, Leeming DJ, Karsdal MA, Krag A, Barrera F, et al Markers of Collagen Remodeling Detect Clinically Significant Fibrosis in Chronic Hepatitis C Patients. PLoS One [Internet]. 2015 1 [cited 2015 Dec 16];10(9):e0137302 Available from: http://www.pubmedcentral.nih.gov/articlerender.fcgi?artid=4583995&tool=pmcentrez&rendertype=abstract 10.1371/journal.pone.0137302 PMC4583995 26406331

[30] van Haaften WT, Mortensen JH, Karsdal MA, Bay-Jensen AC, Dijkstra G, Olinga P. Misbalance in type III collagen formation/degradation as a novel serological biomarker for penetrating (Montreal B3) Crohn’s disease. Aliment Pharmacol Ther. 2017;46(1):26–39. doi: 10.1111/apt.14092 28481042 10.1111/apt.14092 PMC6221070

[31] Leeming DJ, Nielsen MJ, Dai Y, Veidal SS, Vassiliadis E, Zhang C, et al Enzyme-linked immunosorbent serum assay specific for the 7S domain of collagen type IV (P4NP 7S): A marker related to the extracellular matrix remodeling during liver fibrogenesis. Hepatol Res. 2012;42(5):482–93. doi: 10.1111/j.1872-034X.2011.00946.x 22221767 10.1111/j.1872-034X.2011.00946.x

[32] Veidal SS, Karsdal MA, Nawrocki A, Larsen MR, Dai Y, Zheng Q, et al Assessment of proteolytic degradation of the basement membrane: a fragment of type IV collagen as a biochemical marker for liver fibrosis. Fibrogenesis Tissue Repair [Internet]. 2011;4(1):22 Available from: http://fibrogenesis.biomedcentral.com/articles/10.1186/1755-1536-4-22 10.1186/1755-1536-4-22 PMC3204229 21970406

[33] Willumsen N, Bager CL, Leeming DJ, Smith V, Karsdal MA, Dornan D, et al Extracellular matrix specific protein fingerprints measured in serum can separate pancreatic cancer patients from healthy controls. Vol. 13, BMC.Cancer. 2013 p. 554-. 10.1186/1471-2407-13-554 PMC4222497 24261855

[34] Sand JMB, Knox AJ, Lange P, Sun S, Kristensen JH, Leeming DJ, et al Accelerated extracellular matrix turnover during exacerbations of COPD. Respir Res [Internet]. 2015;16(1):69 Available from: http://respiratory-research.com/content/16/1/69 10.1186/s12931-015-0225-3 PMC4491243 26062683

[35] Glover SJ, Garnero P, Naylor K, Rogers A, Eastell R. Establishing a reference range for bone turnover markers in young, healthy women. Bone [Internet]. 2008 4 [cited 2017 Jun 22];42(4):623–30. Available from: http://linkinghub.elsevier.com/retrieve/pii/S8756328207011088 10.1016/j.bone.2007.12.218 18289953

[36] Mart?nez J, Olmos JM, Hern?ndez JL, Pinedo G, Llorca J, Obreg?n E, et al Bone turnover markers in Spanish postmenopausal women. Clin Chim Acta [Internet]. 2009 11 [cited 2017 Jun 22];409(1–2):70–4. Available from: http://www.ncbi.nlm.nih.gov/pubmed/19737549 10.1016/j.cca.2009.08.020 19737549

[37] Morovat A, Catchpole A, Meurisse A, Carlisi A, Bekaert A-C, Rousselle O, et al IDS iSYS automated intact procollagen-1-N-terminus pro-peptide assay: method evaluation and reference intervals in adults and children. Clin Chem Lab Med [Internet]. 2013 10 1 [cited 2017 Jun 22];51(10):2009–18. Available from: https://www.degruyter.com/view/j/cclm.2013.51.issue-10/cclm-2012-0531/cclm-2012-0531.xml 10.1515/cclm-2012-0531 24072575

[38] Danne T, Grüters A, Schuppan D, Quantas N, Enders I, Weber B. Relationship of procollagen type III propeptide-related antigens in serum to somatic growth in healthy children and patients with growth disorders. J Pediatr [Internet]. 1989 2 [cited 2017 Jun 22];114(2):257–60. Available from: http://www.ncbi.nlm.nih.gov/pubmed/2915284 10.1016/s0022-3476(89)80792-9 2915284

[39] Trivedi P, Cheeseman P, Portmann B, Hegarty J, Mowat AP. Variation in serum type III procollagen peptide with age in healthy subjects and its comparative value in the assessment of disease activity in children and adults with chronic active hepatitis. Eur J Clin Invest [Internet]. 1985 4 [cited 2017 Jun 22];15(2):69–74. Available from: http://www.ncbi.nlm.nih.gov/pubmed/3922770 10.1111/j.1365-2362.1985.tb00147.x 3922770

[40] Daan NMP, Fauser BCJM. Menopause prediction and potential implications. Vol. 82, Maturitas. 2015 p. 257–65. 10.1016/j.maturitas.2015.07.019 26278873

[41] Green J, Schotland S, Stauber DJ, Kleeman CR, Clemens TL. Cell-matrix interaction in bone: type I collagen modulates signal transduction in osteoblast-like cells. Am J Physiol [Internet]. 1995;268(5 Pt 1):C1090–103. Available from: http://www.ncbi.nlm.nih.gov/entrez/query.fcgi?cmd=Retrieve&db=PubMed&dopt=Citation&list_uids=7762601 10.1152/ajpcell.1995.268.5.C1090 7762601

[42] Nielsen MJ, Kazankov K, Leeming DJ, Karsdal MA, Krag A, Barrera F, et al Markers of collagen remodeling detect clinically significant fibrosis in chronic hepatitis C patients. PLoS One. 2015;10(9). 10.1371/journal.pone.0137302 PMC4583995 26406331

[43] Eastell R, Szulc P. Use of bone turnover markers in postmenopausal osteoporosis. Lancet Diabetes Endocrinol [Internet]. 2017; Available from: http://linkinghub.elsevier.com/retrieve/pii/S2213858717301845 10.1016/S2213-8587(17)30184-5 28689768

[44] Karsdal MA, Henriksen K, Nielsen MJ, Byrjalsen I, Leeming DJ, Gardner S, et al Fibrogenesis assessed by serological type III collagen formation identifies patients with progressive liver fibrosis and responders to a potential antifibrotic therapy. Am J Physiol—Gastrointest Liver Physiol [Internet]. 2016 12 1 [cited 2017 Aug 10];311(6):G1009–17. Available from: http://www.ncbi.nlm.nih.gov/pubmed/27765759 10.1152/ajpgi.00283.2016 27765759

[45] Bonnans C, Chou J, Werb Z. Remodelling the extracellular matrix in development and disease. Vol. 15, Nat.Rev.Mol.Cell Biol. p. 786–801. 10.1038/nrm3904 PMC4316204 25415508

[46] Lu P, Weaver VM, Werb Z. The extracellular matrix: a dynamic niche in cancer progression. Vol. 196, Journal of Cell Biology. p. 395–406. 10.1083/jcb.201102147 PMC3283993 22351925

[47] Barcellos-Hoff MH, Lyden D, Wang TC. The evolution of the cancer niche during multistage carcinogenesis. Nat Rev Cancer [Internet]. 2013 6 13 [cited 2017 Aug 10];13(7):511–8. Available from: http://www.ncbi.nlm.nih.gov/pubmed/23760023 10.1038/nrc3536 23760023

[48] Langley RR, Fidler IJ. The seed and soil hypothesis revisited—the role of tumor-stroma interactions in metastasis to different organs. Int J cancer [Internet]. 2011 6 1 [cited 2017 Aug 10];128(11):2527–35. Available from: http://doi.wiley.com/10.1002/ijc.26031 10.1002/ijc.26031 PMC3075088 21365651

[49] Bihlet AR, Karsdal MA, Bay-Jensen A-C, Read S, Kristensen JH, Sand JMB, et al Clinical Drug Development Using Dynamic Biomarkers to Enable Personalized Health Care in COPD. Chest [Internet]. 2015 7 [cited 2017 Aug 10];148(1):16–23. Available from: http://www.ncbi.nlm.nih.gov/pubmed/25856563 10.1378/chest.15-0296 25856563

[50] Karsdal MA, Bay-Jensen A-C, Henriksen K, Christiansen C, Genant HK, Chamberlain C, et al Rheumatoid Arthritis: A Case for Personalized Health Care? Arthritis Care Res (Hoboken) [Internet]. 2014 9 [cited 2017 Aug 10];66(9):1273–80. Available from: http://www.ncbi.nlm.nih.gov/pubmed/24470057 10.1002/acr.22289 24470057

