# Supplementary material for: Age-related collagen turnover of the interstitial matrix and basement membrane: Implications of age- and sex-dependent remodeling of the extracellular matrix
Source: PLoS One. 2018 Mar 29;13(3):e0194458. doi: 10.1371/journal.pone.0194458 (PMC5875766; doi:10.1371/journal.pone.0194458)
Supplement: S1 Table — Statistical significance (p-value) for each biomarker calculated using ANOVA comparing the mean of each group with the mean of every other group. Ns refers to no significance. The following biomarkers showed no significance and are therefore not presented: C1M (men), C3M (men), P4NP7S (men and women) and C4M (men). (DOCX) [file pone.0194458.s001.docx]

**Supplementary – table S1**

| **PINP - men** |  |  |  |  |  |  |  |  |  |  |  |  |
| --- | --- | --- | --- | --- | --- | --- | --- | --- | --- | --- | --- | --- |
|  |  |  |  |  |  |  |  |  |  |  |  |  |
| **Age** | **25-29** | **30-34** | **35-39** | **40-44** | **45-49** | **50-54** | **55-59** | **60-64** | **65-69** | **70-74** | **75-79** | **80+** |
| **20-24** | ns | ns | ns | ns | 0.0032 | 0.0056 | 0.0001 | <0.0001 | <0.0001 | <0.0001 | 0.0021 | 0.0003 |
| **25-29** |  | ns | ns | ns | 0.0054 | 0.0091 | 0.0002 | <0.0001 | <0.0001 | <0.0001 | 0.0035 | 0.0004 |
| **30-34** |  |  | ns | ns | ns | ns | 0.017 | <0.0001 | 0.0002 | 0.0002 | ns | 0.0333 |
| **35-39** |  |  |  | ns | ns | ns | ns | 0.0067 | ns | ns | ns | ns |
| **40-44** |  |  |  |  | ns | ns | ns | 0.0348 | ns | ns | ns | ns |
| **45-49** |  |  |  |  |  | ns | ns | ns | ns | ns | ns | ns |
| **50-54** |  |  |  |  |  |  | ns | ns | ns | ns | ns | ns |
| **55-59** |  |  |  |  |  |  |  | ns | ns | ns | ns | ns |
| **60-64** |  |  |  |  |  |  |  |  | ns | ns | ns | ns |
| **65-69** |  |  |  |  |  |  |  |  |  | ns | ns | ns |
| **70-74** |  |  |  |  |  |  |  |  |  |  | ns | ns |
| **75-79** |  |  |  |  |  |  |  |  |  |  |  | ns |

| **PINP - women** | |  |  |  |  |  |  |  |  |  |  |  |
| --- | --- | --- | --- | --- | --- | --- | --- | --- | --- | --- | --- | --- |
|  |  |  |  |  |  |  |  |  |  |  |  |  |
| **Age** | **25-29** | **30-34** | **35-39** | **40-44** | **45-49** | **50-54** | **55-59** | **60-64** | **65-69** | **70-74** | **75-79** | **80+** |
| **20-24** | ns | ns | ns | ns | ns | ns | ns | ns | ns | ns | ns | ns |
| **25-29** |  | ns | ns | ns | ns | ns | ns | ns | ns | ns | ns | ns |
| **30-34** |  |  | ns | ns | ns | ns | ns | ns | ns | ns | ns | ns |
| **35-39** |  |  |  | ns | ns | ns | ns | 0.003 | 0.0254 | ns | ns | 0.0084 |
| **40-44** |  |  |  |  | ns | ns | ns | 0.0247 | ns | ns | ns | ns |
| **45-49** |  |  |  |  |  | ns | ns | 0.0165 | ns | ns | ns | 0.0367 |
| **50-54** |  |  |  |  |  |  | ns | ns | ns | ns | ns | ns |
| **55-59** |  |  |  |  |  |  |  | ns | ns | ns | ns | ns |
| **60-64** |  |  |  |  |  |  |  |  | ns | ns | ns | ns |
| **65-69** |  |  |  |  |  |  |  |  |  | ns | ns | ns |
| **70-74** |  |  |  |  |  |  |  |  |  |  | ns | ns |
| **75-79** |  |  |  |  |  |  |  |  |  |  |  | ns |

| **C1M - women** | |  |  |  |  |  |  |  |  |  |  |  |
| --- | --- | --- | --- | --- | --- | --- | --- | --- | --- | --- | --- | --- |
|  |  |  |  |  |  |  |  |  |  |  |  |  |
| **Age** | **25-29** | **30-34** | **35-39** | **40-44** | **45-49** | **50-54** | **55-59** | **60-64** | **65-69** | **70-74** | **75-79** | **80+** |
| **20-24** | ns | ns | ns | ns | ns | ns | ns | ns | ns | ns | ns | ns |
| **25-29** |  | ns | ns | ns | ns | ns | ns | ns | ns | ns | ns | ns |
| **30-34** |  |  | ns | ns | ns | ns | ns | ns | ns | ns | ns | ns |
| **35-39** |  |  |  | ns | ns | ns | ns | ns | ns | ns | ns | ns |
| **40-44** |  |  |  |  | ns | ns | ns | ns | ns | 0.0095 | ns | ns |
| **45-49** |  |  |  |  |  | ns | ns | ns | ns | ns | ns | ns |
| **50-54** |  |  |  |  |  |  | ns | ns | ns | ns | ns | ns |
| **55-59** |  |  |  |  |  |  |  | ns | ns | ns | ns | ns |
| **60-64** |  |  |  |  |  |  |  |  | ns | ns | ns | ns |
| **65-69** |  |  |  |  |  |  |  |  |  | ns | ns | ns |
| **70-74** |  |  |  |  |  |  |  |  |  |  | ns | ns |
| **75-79** |  |  |  |  |  |  |  |  |  |  |  | ns |

| **Pro-C3 - men** |  |  |  |  |  |  |  |  |  |  |  |  |
| --- | --- | --- | --- | --- | --- | --- | --- | --- | --- | --- | --- | --- |
|  |  |  |  |  |  |  |  |  |  |  |  |  |
| **Age** | **25-29** | **30-34** | **35-39** | **40-44** | **45-49** | **50-54** | **55-59** | **60-64** | **65-69** | **70-74** | **75-79** | **80+** |
| **20-24** | ns | ns | ns | ns | 0.0289 | ns | ns | ns | ns | ns | ns | ns |
| **25-29** |  | ns | ns | ns | ns | ns | ns | ns | ns | ns | ns | ns |
| **30-34** |  |  | ns | ns | ns | ns | ns | ns | ns | ns | ns | ns |
| **35-39** |  |  |  | ns | ns | ns | ns | ns | ns | ns | ns | ns |
| **40-44** |  |  |  |  | ns | ns | ns | ns | ns | ns | ns | ns |
| **45-49** |  |  |  |  |  | ns | ns | ns | ns | ns | 0.0045 | 0.0387 |
| **50-54** |  |  |  |  |  |  | ns | ns | ns | ns | 0.0194 | ns |
| **55-59** |  |  |  |  |  |  |  | ns | ns | ns | ns | ns |
| **60-64** |  |  |  |  |  |  |  |  | ns | ns | ns | ns |
| **65-69** |  |  |  |  |  |  |  |  |  | ns | ns | ns |
| **70-74** |  |  |  |  |  |  |  |  |  |  | ns | ns |
| **75-79** |  |  |  |  |  |  |  |  |  |  |  | ns |

| **Pro-C3 - women** | |  |  |  |  |  |  |  |  |  |  |  |
| --- | --- | --- | --- | --- | --- | --- | --- | --- | --- | --- | --- | --- |
|  |  |  |  |  |  |  |  |  |  |  |  |  |
| **Age** | **25-29** | **30-34** | **35-39** | **40-44** | **45-49** | **50-54** | **55-59** | **60-64** | **65-69** | **70-74** | **75-79** | **80+** |
| **20-24** | ns | ns | ns | ns | ns | ns | ns | ns | ns | ns | ns | ns |
| **25-29** |  | ns | ns | ns | ns | ns | ns | ns | ns | ns | ns | ns |
| **30-34** |  |  | ns | ns | ns | ns | ns | ns | ns | ns | ns | ns |
| **35-39** |  |  |  | ns | ns | ns | ns | ns | ns | ns | ns | ns |
| **40-44** |  |  |  |  | ns | ns | ns | ns | ns | ns | ns | ns |
| **45-49** |  |  |  |  |  | ns | ns | ns | ns | ns | ns | 0.0033 |
| **50-54** |  |  |  |  |  |  | ns | ns | ns | ns | ns | ns |
| **55-59** |  |  |  |  |  |  |  | ns | ns | ns | ns | 0.0026 |
| **60-64** |  |  |  |  |  |  |  |  | ns | ns | ns | ns |
| **65-69** |  |  |  |  |  |  |  |  |  | ns | ns | ns |
| **70-74** |  |  |  |  |  |  |  |  |  |  | ns | ns |
| **75-79** |  |  |  |  |  |  |  |  |  |  |  | ns |

| **C3M - women** | |  |  |  |  |  |  |  |  |  |  |  |
| --- | --- | --- | --- | --- | --- | --- | --- | --- | --- | --- | --- | --- |
|  |  |  |  |  |  |  |  |  |  |  |  |  |
| **Age** | **25-29** | **30-34** | **35-39** | **40-44** | **45-49** | **50-54** | **55-59** | **60-64** | **65-69** | **70-74** | **75-79** | **80+** |
| **20-24** | ns | ns | ns | ns | ns | ns | ns | ns | ns | ns | ns | ns |
| **25-29** |  | ns | ns | ns | ns | ns | ns | ns | ns | ns | ns | ns |
| **30-34** |  |  | ns | ns | ns | ns | ns | ns | ns | ns | ns | ns |
| **35-39** |  |  |  | ns | ns | ns | ns | ns | ns | ns | ns | ns |
| **40-44** |  |  |  |  | ns | ns | 0.0128 | ns | ns | ns | ns | ns |
| **45-49** |  |  |  |  |  | ns | 0.0048 | ns | ns | ns | ns | ns |
| **50-54** |  |  |  |  |  |  | ns | ns | ns | ns | ns | ns |
| **55-59** |  |  |  |  |  |  |  | 0.0339 | ns | ns | ns | ns |
| **60-64** |  |  |  |  |  |  |  |  | ns | ns | ns | ns |
| **65-69** |  |  |  |  |  |  |  |  |  | ns | ns | ns |
| **70-74** |  |  |  |  |  |  |  |  |  |  | ns | ns |
| **75-79** |  |  |  |  |  |  |  |  |  |  |  | ns |

| **C4M - women** | |  |  |  |  |  |  |  |  |  |  |  |  |
| --- | --- | --- | --- | --- | --- | --- | --- | --- | --- | --- | --- | --- | --- |
|  |  | |  |  |  |  |  |  |  |  |  |  |  |
| **Age** | **25-29** | | **30-34** | **35-39** | **40-44** | **45-49** | **50-54** | **55-59** | **60-64** | **65-69** | **70-74** | **75-79** | **80+** |
| **20-24** | ns | | ns | ns | ns | ns | ns | ns | ns | ns | ns | ns | ns |
| **25-29** |  | | ns | ns | ns | ns | ns | 0.0011 | ns | ns | ns | ns | ns |
| **30-34** |  | |  | ns | ns | ns | ns | 0.0285 | ns | ns | ns | ns | ns |
| **35-39** |  | |  |  | ns | ns | ns | ns | ns | ns | ns | ns | ns |
| **40-44** |  | |  |  |  | ns | ns | ns | ns | ns | ns | ns | ns |
| **45-49** |  | |  |  |  |  | ns | ns | ns | ns | ns | ns | ns |
| **50-54** |  | |  |  |  |  |  | ns | ns | ns | ns | ns | ns |
| **55-59** |  | |  |  |  |  |  |  | ns | ns | ns | ns | 0.0129 |
| **60-64** |  | |  |  |  |  |  |  |  | ns | ns | ns | ns |
| **65-69** |  | |  |  |  |  |  |  |  |  | ns | ns | ns |
| **70-74** |  | |  |  |  |  |  |  |  |  |  | ns | ns |
| **75-79** |  | |  |  |  |  |  |  |  |  |  |  | ns |
